# Proteolytic cleavage of HLA class II by human neutrophil elastase in pneumococcal pneumonia

**DOI:** 10.1038/s41598-021-82212-5

**Published:** 2021-01-28

**Authors:** Hisanori Domon, Tomoki Maekawa, Toshihito Isono, Kazuyuki Furuta, Chikara Kaito, Yutaka Terao

**Affiliations:** 1 Division of Microbiology and Infectious Diseases, Niigata University Graduate School of Medical and Dental Sciences, Niigata, Japan; 2 Graduate School of Medicine, Dentistry, and Pharmaceutical Sciences, Okayama University, Okayama, Japan

**Keywords:** Microbiology, Bacterial immune evasion, Bacterial toxins, Infectious diseases, Bacterial infection

## Abstract

Bacterial and viral respiratory infections can initiate acute lung injury and acute respiratory distress syndrome. Neutrophils and their granule enzymes, including neutrophil elastase, are key mediators of the pathophysiology of acute respiratory failure. Although intracellular neutrophil elastase functions as a host defensive factor against pathogens, its leakage into airway spaces induces degradation of host connective tissue components. This leakage disrupts host innate immune responses via proteolytic cleavage of Toll-like receptors and cytokines. Here, we investigated whether neutrophils possess proteases that cleave adaptive immune molecules. We found that expression of the human leukocyte antigen (HLA) class II molecule HLA-DP β1 was decreased in THP-1-derived macrophages treated with supernatants from dead neutrophils. This decreased HLA-DP β1 expression was counteracted by treatment with neutrophil elastase inhibitor, suggesting proteolytic cleavage of HLA-DP β1 by neutrophil elastase. SDS-PAGE showed that neutrophil elastase cleaved recombinant HLA-DP α1, -DP β1, -DQ α1, -DQ β1, -DR α, and -DR β1. Neutrophil elastase also cleaved HLA-DP β1 on extracellular vesicles isolated from macrophages without triggering morphological changes. Thus, leakage of neutrophil elastase may disrupt innate immune responses, antigen presentation, and T cell activation. Additionally, inhibition of neutrophil elastase is a potential therapeutic option for treating bacterial and viral pneumonia.

## Introduction

Acute lung injury (ALI) and acute respiratory distress syndrome (ARDS) are life-threatening diseases in critically ill patients and represent a spectrum of progressive respiratory failure affecting approximately 200,000 patients, causing 75,000 deaths annually, in the United States^1^. These conditions are characterized by severe hypoxemia, hypercapnia, diffuse alveolar infiltrates on chest radiographs, and a substantial reduction in pulmonary compliance^2^. Such acute respiratory failure is commonly associated with pulmonary and systemic inflammation, including severe pneumonia, aspirating gastric contents, and sepsis^1,3^. Among these, bacterial pneumonia is considered, particularly that caused by *Streptococcus pneumoniae*, *Haemophilus influenzae*, and *Staphylococcus aureus* as the primary pathogens. Currently, coronaviruses are important causes of respiratory tract infections that can result in ALI and ARDS due to accumulation of neutrophils in lung tissue^4–6^. Although excessive activation of alveolar neutrophils is thought to play a crucial role in these lung diseases, the pathogenicity of these cells has rarely been studied.

Neutrophils are the most abundant leukocytes in the human blood, constituting 50–70% of white blood cells. In response to inflammatory stimulation such as respiratory infections, neutrophils migrate from the bloodstream to the site of infection, where they efficiently phagocytize and rapidly inactivate pathogens using antimicrobial compounds stored in granules^7^. Reportedly, the antibacterial compounds include cathelicidin, lysozyme, lactoferrin, and several serine proteases, including cathepsin G, neutrophil elastase (NE), proteinase 3, and neutrophil serine protease 4^8^.

Among neutrophil serine proteases, NE has been well-studied at both the basic research and clinical practice levels in lung disease. NE degrades outer membrane proteins on the surface of Gram-negative bacteria^9^. For Gram-positive pneumococci, NE plays a crucial role in intracellular bacterial killing by neutrophils following phagocytosis^10^. Although the role of NE during viral infections is not fully understood, elevation of its levels is part of an inflammatory response to multiple viral infections^11–13^. Alternatively, once NE is released into the extracellular space, it may degrade not only pathogens but also host connective tissue components, including elastin, proteoglycan, fibronectin and several collagen types, resulting in tissue destruction^14^. We previously demonstrated that *S. pneumoniae* produces pneumolysin (PLY), a pore-forming toxin, which leads to neutrophil lysis and induces NE leakage^15^. Subsequently, extracellular NE cleaves several host proteins associated with the innate immune response, including pulmonary surfactant protein D^16^, C5a receptor^17^, Toll-like receptors, and proinflammatory cytokines^18^. However, the consequences of leakage of neutrophil serine proteases on the adaptive immune system are not fully understood.

Understanding the physiological activity of granule enzyme and its role may lead to better treatments for acute pulmonary failure. As NE cleaves multiple host cell-surface molecules, we hypothesized that neutrophil cell death promotes the cleavage of major histocompatibility complex (MHC) class II proteins. Therefore, the main objective of this study was to investigate the effect of supernatants from dead neutrophils on MHC class II expression on macrophages.

## Results

### HLA class II expression is decreased on human macrophages treated with culture supernatants from pore-forming toxin-treated neutrophils

**Figure 1:**
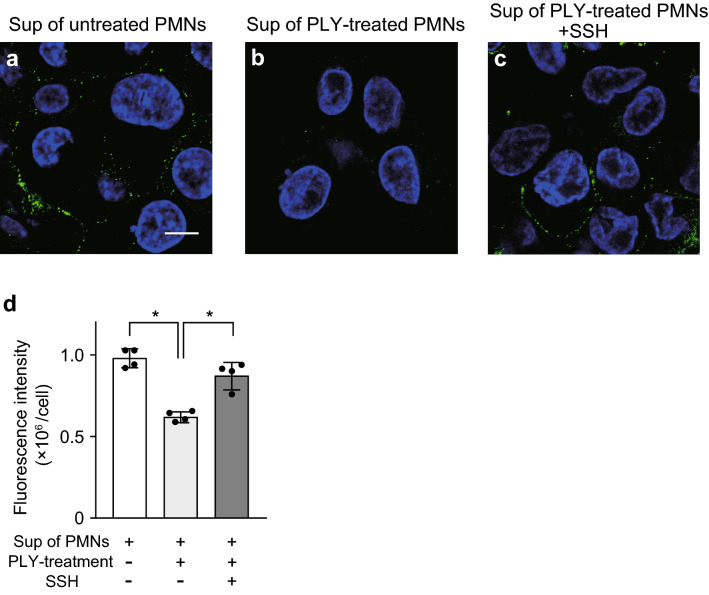
Culture supernatant from neutrophils treated with pore-forming toxin decreases fluorescence intensity of HLA class II on macrophages. (**a**–**c**) THP-1-derived macrophages were exposed to culture supernatant (Sup) from untreated neutrophils (PMNs) or pneumolysin (PLY)-treated PMNs in the presence or absence of 100 μg/mL sivelestat sodium hydrate (neutrophil elastase inhibitor; SSH) for 6 h. Representative fluorescence microscopy images of macrophages stained for DNA (DAPI; blue) and HLA class II (HLA-DP β1; green) are shown. Scale bar: 10 µm. (**d**) Fluorescence intensity of HLA class II per cell was calculated. Data representing the mean ± SD of quadruplicate experiments was evaluated by one-way analysis of variance with Tukey’s multiple-comparisons test. *Significantly different between the indicated groups at *P* < 0.01.

We first investigated whether neutrophils leak proteases that cleave HLA class II. Accordingly, primary isolated human neutrophils were pretreated with PLY; the supernatant from dead neutrophils was added to THP-1-derived macrophages (hereafter designated as macrophages). The fluorescence intensity of HLA-DP β1, a class II β-chain, was decreased in macrophages treated with supernatants from dead neutrophils compared to after treatment with supernatants from untreated neutrophils (Fig. 1a,b). We hypothesized that NE leakage induces cleavage of HLA class II and investigated the effect of the NE inhibitor sivelestat sodium hydrate (SSH) on HLA-DP β1 expression. As shown in Fig. 1c,d, the decrease in fluorescence intensity of HLA-DP β1 produced by the dead-neutrophil supernatants was counteracted by SSH-treatment.

### Human neutrophil elastase (hNE) cleaves HLA class II on human macrophages

**Figure 2:**
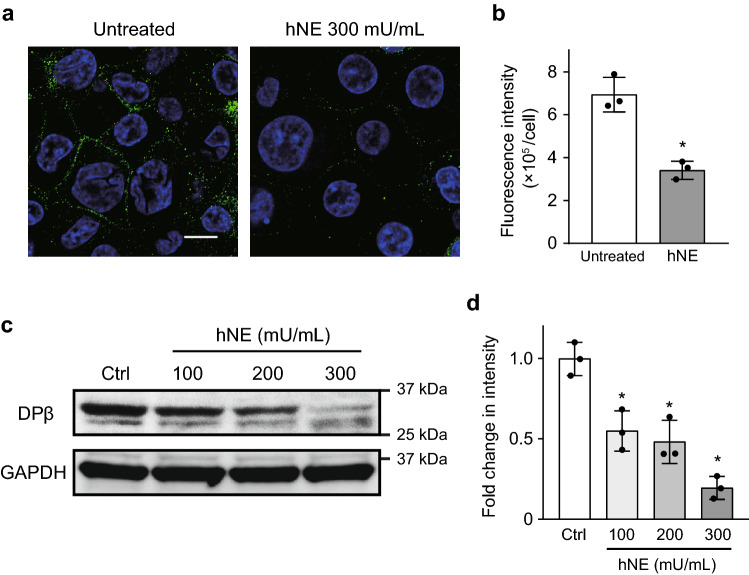
Neutrophil elastase (NE) decreases the fluorescence intensity of HLA class II on macrophages. THP-1-derived macrophages were cultured in RPMI 1640 and exposed to hNE (100–300 mU/mL) for 6 h. (**a**) Representative fluorescence microscopy images of untreated and 300 mU/mL hNE-treated macrophages stained for DNA (DAPI; blue) and HLA class II (HLA-DP β1; green) are shown. Scale bar: 10 µm. (**b**) The fluorescence intensity of HLA class II per cell was calculated. Data represent the mean ± SD of triplicate experiments and were evaluated using unpaired *t* tests. *Significantly different from the untreated control group at *P* < 0.01. (**c**) HLA-DP β1 expression in macrophages was determined by Western blot analysis. (**d**) Intensity of Western blotting signals of HLA-DP β1 in macrophage was quantified by densitometry. Data represent the means ± SD of triplicate experiments and were evaluated by one-way analysis of variance with Dunnett’s multiple-comparisons test. *Significantly different as compared with that of the control group at *P* < 0.01.

Our previous study demonstrated that NE activity was elevated in the bronchoalveolar lavage fluid (BALF) of a murine model of intratracheal pneumococcal infection compared to in uninfected controls (average 431.6 vs. 2.6 mU/mL)^18^. Thus, we treated human macrophages with up to 300 mU/mL of hNE. Immunofluorescence confocal microscopy and Western blotting indicated that 300 mU/mL hNE significantly decreased the intensity of HLA-DP β1 on macrophages (Fig. 2a–d). However, treatment with 300 mU/mL hNE affected neither gene transcription of HLA class II-related genes (*HLA-DPB1*, *HLA-DQB1*, and *HLA-DRA*; Supplementary Fig. S1 online) nor cell viability in macrophages^18^. Overall, our findings suggest that hNE in the supernatant from PLY-treated neutrophils cleaves HLA-DP β1 on macrophages.

### hNE cleaves not only HLA-DP β1, but also HLA-DQ and HLA-DR

**Figure 3:**
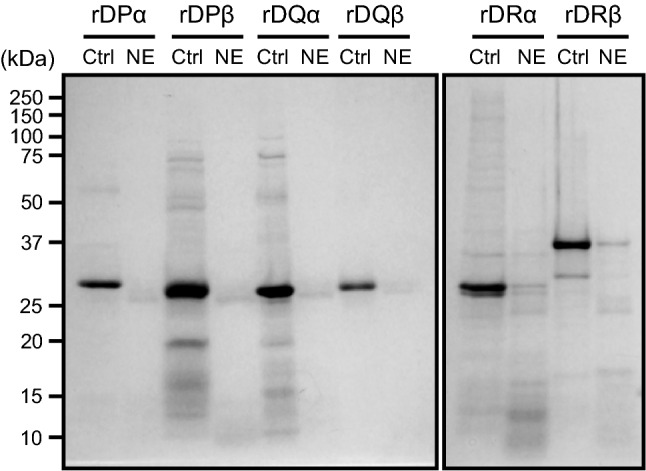
Neutrophil elastase (NE) degrades recombinant proteins which constitute HLA class II. Recombinant (r) HLA-DP α1, rHLA-DP β1, rHLA-DQ α1, rHLA-DQ β1, rHLA-DR α, and rHLA-DR β1 were treated with 300 mU/mL hNE at 37 °C for 3 h. All samples were separated by SDS-PAGE followed by Coomassie brilliant blue staining.

HLA class II genes are composed of three closely linked subregions encoding the polymorphic HLA class II molecules HLA-DP, -DQ, and -DR^19^. We investigated whether hNE cleaves recombinant (r) HLA class II molecules rHLA-DP α1, -DP β1, -DQ α1, -DQ β1, -DR α, and -DR β1. SDS-PAGE showed that all of these molecules had almost disappeared after 3-h treatment with 300 mU/mL NE (Fig. 3). Additionally, rHLA-DP β1, -DR α, and -DR β1 exhibited lower-molecular-mass fragments upon hNE treatment. These findings indicate that hNE cleaves multiple HLA class II molecules, which in turn results in decreased HLA class II expression on macrophages.

### hNE cleaves HLA class II on extracellular vesicles

**Figure 4:**
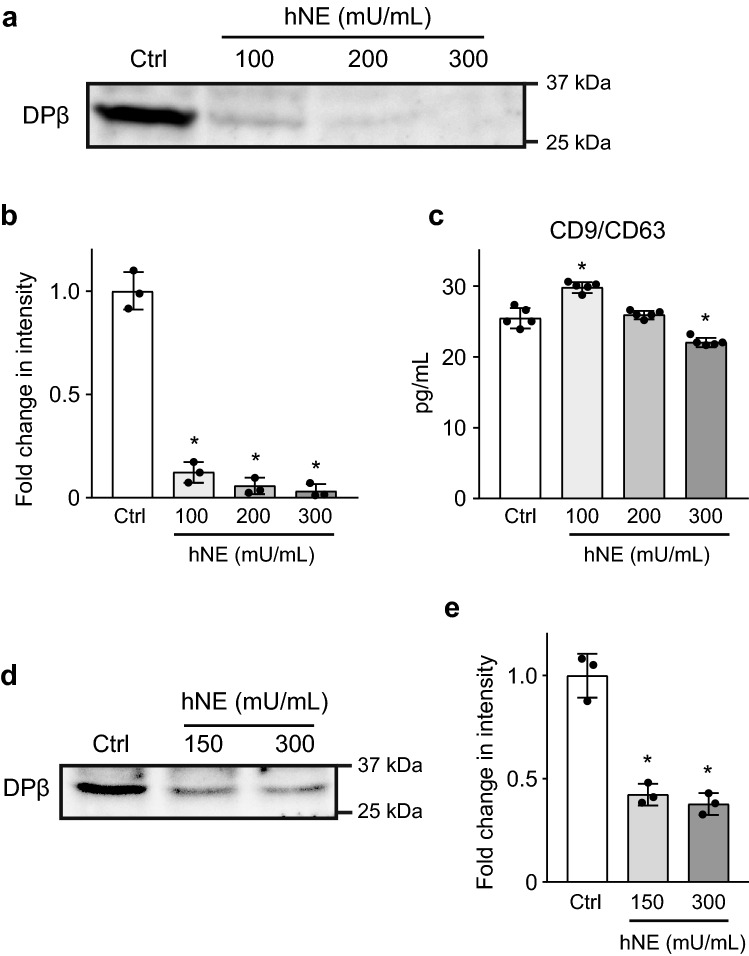
Neutrophil elastase (NE) degrades HLA class II on extracellular vesicles derived from macrophages. (**a**) THP-1-derived macrophages were cultured in RPMI 1640 in the presence or absence of various concentrations of hNE (100–300 mU/mL) for 6 h. HLA-DP β1 expression on extracellular vesicles isolated from supernatant samples was determined by Western blot analysis. (**b**) Intensity of Western blotting signals of HLA-DP β1 on extracellular vesicles isolated from supernatant samples was quantified by densitometry. Data represent the means ± SD of triplicate experiments and were evaluated by one-way analysis of variance with Dunnett’s multiple-comparisons test. *Significantly different as compared with that of the control group at *P* < 0.01. (**c**) Extracellular vesicle production in supernatant samples were measured with a CD9/CD63 ELISA kit. Data represent the mean ± SD of quintuplicate experiments and were evaluated by one-way analysis of variance with Dunnett's multiple-comparison test. *Significantly different from control group at *P* < 0.01. (**d**) Extracellular vesicles were isolated from supernatant of THP-1 derived macrophages followed by hNE-treatment (150 and 300 mU/mL) for 4 h. Western blot analysis was performed to determine HLA-DP β1 expression in NE-treated and untreated extracellular vesicles. (**e**) Intensity of Western blotting signals of HLA-DP β1 on hNE-treated extracellular vesicles was quantified by densitometry. Data represent the means ± SD of triplicate experiments and were evaluated by one-way analysis of variance with Dunnett’s multiple-comparisons test. *Significantly different as compared with that of the control group at *P* < 0.01.

Reportedly, antigen-presenting cells (APCs) secrete exosomes that carry peptide-loaded HLA molecules that can stimulate T-cell proliferation in vitro^20,21^. In addition, exosomes, which express HLA class II and co-stimulatory molecules, are present in the BALF obtained from healthy individuals, suggesting that they play a role in antigen delivery or immune regulation during airway antigen exposure^22^. Based on this, we examined whether hNE-treatment affects HLA class II expression on extracellular vesicles. Supplementary Fig. S2 shows the characterization of extracellular vesicles isolated from macrophages. The mean particle diameter was 134 ± 2.8 nm (mode: 103 ± 3.7 nm) (Supplementary Fig. S2a online). The extracellular vesicles expressed multiple exosome markers, including CD63, CD81, ALIX, FLOT1, ICAM1, EpCAM, ANXA5, and TSG101. However, the expression of GM130, a marker of cellular contamination, was minimal (Supplementary Fig. S2b online). Figures 4a,b show that the protein bands representing HLA-DP β1 on extracellular vesicles isolated from hNE-treated macrophages were significantly decreased in intensity relative to those from untreated macrophages. Enzyme-linked immunosorbent assay (ELISA) demonstrated that the concentration of extracellular vesicles was only marginally decreased in the supernatant from 300 mU/mL hNE-treated macrophages (Fig. 4c). These data suggest two hypotheses: (i) hNE directly cleaves HLA-DP β1 on extracellular vesicles; (ii) hNE-induced cleavage of HLA-DP β1 on macrophages results in release of extracellular vesicles that express lower levels of the molecule. To clarify the effect of hNE on HLA-DP β1 expression on extracellular vesicles, we isolated extracellular vesicles from untreated macrophages and then exposed the vesicles to hNE followed by Western blotting with anti- HLA-DP β1 antibody. Figures 4d,e shows that the intensity of protein bands significantly decreased after hNE-treatment, indicating that hNE directly cleaves HLA class II on extracellular vesicles. Treatment with hNE did not induce obvious morphological changes (Supplementary Fig. S3 online).

### Intratracheal infection with pneumococcus increases the level of murine MHC class II in cell-free BALF

**Figure 5:**
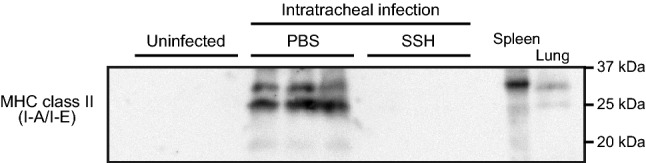
Murine MHC class II fragments are detectable in cell-free bronchoalveolar lavage fluid (BALF) from a mouse model of pneumococcal intratracheal infection. BALB/c mice (n = 7) were intratracheally infected with *Streptococcus pneumoniae* D39 (2 × 10^8^ colony forming units in 50 µL PBS). Uninfected mice were administered PBS only. Neutrophil elastase (NE) inhibitor (sivelestat sodium hydrate (SSH) group; 50 mg/kg) or PBS (PBS group) was administered intraperitoneally into the infected mice every 6 h. MHC class II (I-A/I-E) levels in the cell-free BALF were determined by Western blotting. Murine spleen and lung tissue lysate from uninfected mice were included as controls. A representative Western blot image (three samples in each group) is shown.

To confirm whether NE induces cleavage of MHC class II in vivo, we investigated the effect of NE on BALB/c mice upon intratracheal infection with pneumococcus in the presence or absence of SSH. Figure 5 shows that the pneumococcal infection facilitated the detection of two isoforms of MHC class II (I-A/I-E) (32 and 24 kDa) with a lower molecular mass fragment (20 kDa) in the cell-free BALF. However, administration of SSH did not cause MHC class II fragmentation. Reportedly, anti-MHC class II (I-A/I-E) antibody (clone: M5/114) reacts with mouse MHC class II haplotypes I-A^d^ and I-E^d^ in BALB/c mice, whereas the antibody does not cross-react with I-A^s^ in SJL/J mice^23^. To determine whether the antibody nonspecifically detects other proteins such as pneumococcal proteins, BALF from SJL/J mice upon intratracheal infection with pneumococcus were also analyzed by immunoblotting. Supplementary Fig. S4 shows that the anti-MHC class II antibody was not specific for BALF from SJL/J mice, indicating the high specificity of the antibody. These findings suggest that murine (m) NE cleaves mMHC class II, resulting in detection of MHC class II fragments in the BALF from a model of intratracheal pneumococcal infection.

## Discussion

Pulmonary host defense responses involve both innate and adaptive immune responses. Innate defenses consist of structural defenses, antimicrobial molecules, and phagocytic defenses provided by resident alveolar macrophages and neutrophils that are recruited into the lung in response to infection^24^. In our previous studies, we demonstrated that *S. pneumoniae* PLY induces neutrophil lysis and NE leakage, which results in disruption of alveolar epithelial cells, impaired phagocytic activity in macrophages, and proteolytic cleavage of innate immune components, including Toll-like receptors and proinflammatory cytokines^15,18^. Here, we addressed the intriguing possibility that at least some adaptive immune molecules, such as HLA class II molecules, on APCs and extracellular vesicles are cleaved by hNE, which was originally identified as a major granule enzyme contributing to host defense against bacterial infection. Overall, leakage of hNE may disrupt not only innate immune responses, but also antigen presentation and T-cell activation.

The adaptive immune system is distinguished by its ability to respond specifically to an almost limitless number of antigens. A key requirement for generating such immune responses involves the display of antigenic peptides on the cell surface by MHC class I and class II. Subsequently, antigen/MHC complexes induce the activation, expansion, and differentiation of T cells into multiple effectors^25^. In addition to neutrophils, T cells rapidly infiltrate into areas of inflamed lung tissue following infection of *S. pneumoniae*^26^. Additionally, MHC class II knockout mice in a model of pneumococcal pneumonia and septicemia showed increased bacterial colony-forming units in the lung tissue and blood, respectively, compared with their wild-type counterparts. A recent study showed that patients with coronavirus disease 2019 pneumonia who developed ARDS exhibited significantly higher neutrophil counts compared to those without ARDS^27^. The study also suggested that higher CD4^+^ T cell counts protect patients from ARDS. These reports indicate the importance of antigen presentation and subsequent CD4^+^ T-cell activation for inducing protective immunity against bacterial and viral pneumonia^28^. Therefore, hNE leakage-induced cleavage of HLA molecules may inhibit CD4^+^ T-cell activation, downregulate cytokine production, and exacerbate severe pneumonia associated with ALI and ARDS. Furthermore, we previously showed that hNE degrades multiple cytokines, including interferon gamma, interleukin (IL)-2, IL-4, and IL-12, suggesting that hNE attenuates multiple adaptive immune responses^18^.

These in vitro findings prompted us to test whether APCs pretreated with hNE impair T-cell activation. However, contrary to the hypothesis, coculture of hNE-pretreated mouse bone marrow dendritic cells with 3A9 T cells showed increased IL-2 in the supernatant compared with in the untreated control (Supplementary Fig. S5a online). Flow cytometry showed that hNE treatment decreased the expression of mouse PD-L1 and PD-L2 on dendritic cells (Supplementary Fig. S5b online), suggesting that hNE cleaves only co-inhibitory molecules on murine APCs, followed by increased activation of T cells. In this context, we previously reported that hNE cleaves human tumor necrosis factor, although murine tumor necrosis factor was not cleaved by hNE in vitro^18^. However, an in vivo mouse intratracheal infection model suggested that mNE cleaves mMHC class II. These results indicate a molecular basis for distinctions in peptide substrate specificities between hNE and mNE, despite their conserved catalytic triad, His, Asp, and Ser^29^. Kalupov et al. reported that mNE did not cleave hNE-specific substrates^30^. They also identified structural differences between mNE and hNE that may account for their differences in substrate specificity. Therefore, there are limitations to using murine cell lines and murine infection models to evaluate the molecular harmfulness of NE in human inflammatory diseases.

In the present study, hNE directly cleaved HLA class II on extracellular vesicles and the intensity of the protein band decreased to approximately 40% in Fig. 4d,e. However, Fig. 4a,b show that HLA-DP β1 expression on extracellular vesicles isolated from hNE-treated macrophages were almost completely absent. These data suggest that hNE-induced cleavage of HLA class II on macrophages may result in release of extracellular vesicles that express lower levels of the molecule. It has been reported that newly synthesized MHC class II complexes are transferred from *trans*-Golgi network to the plasma membrane^31^. Following endocytosis, MHC class II complexes are internalized and traffic to antigen-processing compartments, which contain intraluminal vesicles^32^. Some of MHC class II complexes are sorted into the intraluminal vesicles. Thereafter, the vesicles are released into the extracellular space and secreted vesicles are termed extracellular vesicles^33^. These observations indicate that, following exposure to hNE, MHC class II complexes are sorted into the vesicles during lung infection. Therefore, it is possible that hNE-mediated cleavage of MHC class II on macrophages results in the decreased MHC class II expression on extracellular vesicles.

Several studies investigated the potential role of various NE inhibitors, including sivelestat, in bacterial pneumonia^34^. In murine models of severe pneumococcal pneumonia, the administration of sivelestat resulted in higher survival rates and decreased bacterial counts in the blood^18,35^. These data suggest that NE-induced lung injury and immune subversion result in bacterial invasion of the bloodstream. Furthermore, the number of *S. pneumoniae* in BALF was also decreased by sivelestat treatment both in murine and hamster models of pneumonia^18,36^. Although there is a possibility that intracellular pneumococcal killing in neutrophils may be partially suppressed by NE inhibition^10^, impairment of phagocytic activity in macrophage and disruption of pulmonary epithelial barrier induced by extracellular NE is counteracted by sivelestat^15^. Taken together, in addition to antibiotic treatment, NE inhibitor is a potential therapeutic option for treating pneumococcal pneumonia.

In conclusion, our data suggest that hNE leakage disrupts host adaptive immunity. Generally, hNE-induced proteolytic degradation of host tissues is inhibited by serum α1-antitrypsin, a serine protease inhibitor. However, in response to microbial invasion, neutrophils also release matrix metalloproteinases that degrade and inactivate α1-antitrypsin, increasing proteolytic activity of hNE, causing ALI^37^. Collectively, these observations suggest that improved therapeutic strategies for pneumonia require inhibition of elastase activity in the lung.

## Methods

### Reagents

Recombinant human proteins used in this study are described in the supporting information. The expression and purification of His-tagged recombinant PLY was previously described^15^.

### Human neutrophil isolation and supernatant preparation

Neutrophils were prepared as previously described^38^. Briefly, heparinized blood obtained from a healthy donor was layered onto Polymorphprep (Axis Shield, Dundee, UK) in a ratio of 1:1 and centrifuged at 500 *g* for 30 min. Layers containing neutrophils were carefully collected and residual red blood cells were lysed by ACK Lysing Buffer (Lonza, Basel, Switzerland). Human neutrophils (1 × 10^7^ cells in 400 μL) were cultured in the presence or absence of recombinant PLY (10 μg/mL) for 6 h and supernatant samples were used for subsequent experiments. The experimental protocol abides by the Declaration of Helsinki principles and was approved by the Institutional Review Board of Niigata University. Experiments were carried out in accordance with approved guidelines. Informed consent was obtained from all donors prior to their inclusion in this study (permit # 2018–0075).

### Cell lines

The monocytic cell line THP-1 was maintained in Roswell Park Memorial Institute (RPMI) 1640 medium supplemented with 10% fetal bovine serum, 100 U/mL penicillin, and 100 µg/mL streptomycin (FUJIFILM Wako Pure Chemical Corporation, Osaka, Japan) at 37 °C in 95% air and 5% CO_2_. The cells were incubated in a multi-well glass-bottom dish (Matsunami Glass, Osaka, Japan) at a concentration of 1 × 10^5^ cells/100 μL in medium supplemented with 200 nM phorbol 12-myristate 13-acetate (Cayman Chemical, Ann Arbor, MI, USA) to induce differentiation into macrophage-like cells. Following 48-h incubation, the cells were washed with serum-free RPMI 1640 and cultured a further 12 h. Thereafter, cells were exposed to supernatant from human neutrophils or 100–300 mU/mL hNE (Innovative Research, Novi, MI, USA) in the presence or absence of SSH (100 µg/mL; ONO Pharmaceutical Co., Osaka, Japan) for 6 h under serum-free conditions.

### Immunofluorescence analysis

Macrophages were fixed with 4% paraformaldehyde for 15 min, followed by incubation of the cells in blocking buffer (Thermo Fisher Scientific, Waltham, MA, USA) for 30 min. Thereafter, the samples were stained with mouse anti-MHC class II (HLA-DP β1) antibody (Abcam, Cambridge, UK) in blocking buffer. Following overnight incubation at 4 °C, secondary AlexaFluor 488-conjugated goat anti-mouse IgG antibody (Thermo Fisher Scientific) in blocking buffer was added and incubated for 2 h. The samples were washed with phosphate-buffered saline (PBS) and imaged with a confocal laser-scanning microscope (Carl Zeiss, Oberkochen, Germany). Additionally, fluorescence intensity of HLA-DP β1 per cell was calculated by using MetaMorph NX software (Molecular Devices, Sunnyvale, CA, USA).

### Proteolytic cleavage analysis

Whole-cell lysates of hNE-treated or untreated macrophages were prepared in Protein Extraction Reagent (Thermo Fisher Scientific). Enrichment of extracellular vesicles from the culture supernatant of macrophages is described in the supplementary information. The samples were mixed with SDS-sample buffer, separated by SDS-PAGE, and transferred to polyvinylidene difluoride membranes (Merck KGaA, Darmstadt, Germany), followed by incubation with blocking reagent (Nacalai Tesque, Kyoto, Japan). The membrane was probed with an anti-MHC class II (HLA-DP β1) antibody or anti-glyceraldehyde 3-phosphate dehydrogenase antibody (Abcam) and then incubated with a horseradish peroxidase-conjugated secondary antibody (Cell Signaling Technology, Danvers, MA, USA) in Tris-buffered saline containing 0.05% Tween 20. The membrane was treated with horseradish peroxidase substrates (GE Healthcare, Little Chalfont, UK) and analyzed with a chemiluminescence detector (Fujifilm, Tokyo, Japan). The intensity of the signal was quantified using Image Studio software version 5.2 (LI-COR Bioscience, Lincoln, NE, USA).

For the recombinant protein cleavage assay, 400–1000 ng of rHLA-DP α1, rHLA-DP β1, rHLA-DQ α1, rHLA-DQ β1, rHLA-DR α, and rHLA-DR β1 were treated with 300 mU/mL hNE at 37 °C for 3 h and mixed with SDS-sample buffer. All samples were separated by SDS-PAGE followed by Coomassie brilliant blue staining (APRO Science, Tokushima, Japan).

### Quantification of extracellular vesicle levels

The extracellular vesicles level in the macrophage supernatant was determined using the CD9/CD63 human exosome ELISA Kit (Cosmo Bio, Tokyo, Japan).

### Pneumococcal intratracheal infection in vivo

Details of the pneumococcal culture and mouse model of intratracheal pneumococcal infection were described previously^18,39^, and additional information can be found in the supplementary information. All animal experiments were approved by the Institutional Animal Care and Use Committee of Niigata University (Approval number: SA00002). We confirmed that all experiments were performed in accordance with relevant guidelines and regulations.

### Statistical analysis

Data were statistically analyzed by one-way analysis of variance with Tukey’s or Dunnett’s multiple-comparison test using Graph Pad Prism version 7.05 (GraphPad Software, Inc., La Jolla, CA, USA).

## Supplementary Information

Supplementary Information

## Data Availability

All data described are contained within the manuscript.
