# Supplementary material for: Proteolytic cleavage of HLA class II by human neutrophil elastase in pneumococcal pneumonia
Source: Sci Rep. 2021 Jan 28;11:2432. doi: 10.1038/s41598-021-82212-5 (PMC7843615; doi:10.1038/s41598-021-82212-5)
Supplement: Supplementary file 1 — Supplementary Information [file 41598_2021_82212_MOESM1_ESM.docx]

**Supplementary Information**

**Proteolytic cleavage of HLA class II by human neutrophil elastase** **in pneumococcal pneumonia**

Hisanori Domon^1^, Tomoki Maekawa^1^, Toshihito Isono^1^, Kazuyuki Furuta^2^, Chikara Kaito^2^, Yutaka Terao^1^*

^1^Division of Microbiology and Infectious Diseases, Niigata University Graduate School of Medical and Dental Sciences, Niigata, Japan.

^2^Graduate School of Medicine, Dentistry, and Pharmaceutical Sciences, Okayama University, Okayama, Japan.

*Corresponding author: Yutaka Terao

E-mail address: [terao@dent.niigata-u.ac.jp](mailto:terao@dent.niigata-u.ac.jp)

**Supporting Methods**

***Reagents***

Recombinant (r) HLA-DP α1, rHLA-DP β1, rHLA-DQ α1, rHLA-DQ β1, rHLA-DR α, and rHLA-DR β1 were purchased from Signalway Antibody (College Park, MD, USA). *Escherichia coli* LPS (serotype O55: B5) and hen egg lysozyme protein (HEL) were purchased from Sigma-Aldrich (St. Louis, MO, USA). Recombinant mouse GM-CSF, FITC-labeled anti-mouse MHC class II antibody (clone: 11-5.2), FITC-labeled anti-mouse MHC class I antibody (clone: 36-7-5), FITC-labeled anti-mouse CD86 antibody (clone: GL-1), APC-labeled anti-mouse CD11c antibody (clone: N418), PE-labeled anti-mouse PD-L1 antibody (clone: 10F.9G2), and PE-labeled anti-mouse PD-L2 antibody (clone: TY25) were purchased from Biolegend (San Diego, CA, USA). Mouse IL-2 ELISA kit was purchased from Thermo Fisher Scientific (Waltham, MA, USA).

***Isolation of extracellular vesicles and transmission electron microscopy analysis***

The monocytic cell line THP-1 was incubated in 100 mm cell culture dishes (Corning, NY, USA) at a concentration of 2 × 10^7^ cells/mL in medium supplemented with 200 nM phorbol 12-myristate 13-acetate (Cayman Chemical, Ann Arbor, MI, USA) to induce differentiation into macrophage-like cells. Following a 48-h incubation, cells were washed with serum-free RPMI 1640 and cultured further for 12 h. Thereafter, cells were washed again with serum-free RPMI 1640 and incubated for an additional 24 h at 37°C in 95% air and 5% CO_2_. Fifty milliliter of culture supernatant was then centrifuged at 10,000 *g* for 30 min at 4°C to remove large extracellular vesicles and concentrated to approximately 4 mL using Vivaspin 20 (Sartorius Corporate Administration GmbH, Gottingen, Germany) followed by extracellular vesicles isolation using MagCapture Exosome Isolation Kit PS (FUJIFILM Wako Pure Chemical Corporation, Osaka, Japan) according to the manufacturer’s protocol. The quality of the extracellular vesicles was examined using Exo-Check Exosome Antibody Arrays (SBI system biosciences, Palo Alto, CA, USA) following manufacturer’s protocol. The antibody arrays included eight antibodies for known exosome markers (CD63, CD81, ALIX, FLOT1, ICAM1, EpCam, ANXA5, and TSG101) and a GM130 cis-Golgi marker to monitor cellular contamination. Thereafter, the size distribution of extracellular vesicle preparations was analyzed using a NanoSight LM10 (Malvern Panalytical Ltd, Malvern, UK) according to the manufacturer’s instructions. The aliquots were then exposed to 300 mU/mL hNE for 6 h at 37°C. Isolated extracellular vesicles were analyzed using an H-7600 transmission electron microscopy (Hitachi High-Technologies, Tokyo, Japan). This analysis was carried out at Hanaichi UltraStracture Research (Aichi, Japan).

***Intratracheal infection of pneumococcus* in vivo**

Male 10- to 12-week-old BALB/c mice and SJL/J mice were obtained from Nihon CLEA (Tokyo, Japan) and Charles River Laboratories Japan Inc. (Yokohama, Japan), respectively. Mice were maintained under standard conditions in accordance with our institutional guidelines. All animal experiments were approved by the Institutional Animal Care and Use Committee of Niigata University (Approval number: SA00002). *Streptococcus pneumoniae* D39 (NCTC 7466) was grown in tryptic soy broth (Becton Dickinson, Franklin Lakes, NJ, USA). Following mouse anesthesia with isoflurane using an inhalational anesthesia system (Natsume Seisakusho, Tokyo, Japan), the trachea was aseptically exposed and an *S. pneumoniae* inoculum (2 × 10^8^ colony forming units in 50 µL PBS) was administered via a 26-gauge needle. Unchallenged naive mice were administered PBS only. NE inhibitor (50 mg/kg) or PBS was administered intraperitoneally to the infected mice every 6 h. Groups of animals were sacrificed at 18 h postinfection. To obtain bronchoalveolar lavage fluid (BALF), 1.0 mL PBS was instilled into mouse lungs and then slowly aspirated. Thereafter, BALF was centrifuged at 1,500 *g*, and the supernatant was used for subsequent western blotting.

***Western blot analysis***

Ten microliters of cell-free BALF sample was mixed with SDS-sample buffer, separated by SDS-PAGE using 12% polyacrylamide gel (Bio-Rad Laboratories, Hercules, CA, USA), and transferred to polyvinylidene difluoride membranes (Merck Millipore, Burlington, MA, USA). The membranes were probed with the anti-MHC class II (I-A/I-E) antibody (clone: M5/114, Bio X Cell, Lebanon, NH, USA) followed by incubation with an HRP-conjugated secondary antibody (Cell Signaling Technology, Danvers, MA, USA). Thereafter, the membranes were incubated with ECL Select reagent (GE Healthcare, Little Chalfont, UK) and analyzed by a chemiluminescence detector (Fujifilm, Tokyo, Japan).

***Isolation of bone marrow-derived dendritic cells***

Male B10.BR mice were obtained from Japan SLC (Hamamatsu, Japan) and kept in a specific-pathogen-free animal facility at Okayama University. All animal experiments were approved by the Institutional Animal Care and Use Committee at Okayama University. Bone marrow-derived dendritic cells (BMDCs) were prepared following the standard protocol ([1](#_ENREF_1)). Briefly, bone marrow cells were collected from the tibia and femur of mice. The cells were cultured in RPMI 1640 medium containing 10% fetal bovine serum (FBS), 50 µM β-mercaptoethanol, 100 U/mL penicillin, 0.1 mg/mL streptomycin in the presence of 10 ng/mL mouse GM-CSF at 37˚C in 5% CO_2_ for 7 d. The medium was changed every 2 d, and BMDC on day 7 was used for the experiment.

***Antigen presentation assay***

BMDCs were cultured in the presence of HEL protein (1 mg/mL) for 24 h at 37˚C followed by stimulation with 100 ng/mL LPS for additional 24 h. The Cells were collected and treated with human neutrophil elastase (hNE; 300 mU/mL) in RPMI 1640 medium under serum-free condition for 3 h. 3A9 cells (ATCC CRL-3293), a T cell hybridoma reactive against HEL presented by APCs in the context of I-A polypeptides, were maintained in RPMI 1640 medium as described above. Thereafter, BMDCs and 3A9 T cells were mixed at 1:5, 1:10, 1:20 and 1:50 ratios and cultured for 24 h. IL-2 concentration in culture supernatants was measured using an ELISA kit according to the manufacturer’s instructions.

***Measurement of cell surface protein expression level***

BMDCs cocultured with 3A9 T cells were collected and stained with fluorescent-labeled antibodies against MHC class I, MHC class II, CD11c, CD86, PD-L1 and PD-L2 in phosphate-buffered saline containing 2% FBS for 30 min on ice. Cells were washed and cell surface expression of these molecules was analyzed using a flow cytometer (Gallios; Beckman Coulter, Brea, CA, USA).

***Quantitative Real-Time PCR***

THP-1-derived macrophages were exposed to 300 mU/mL hNE for 4 h under serum-free conditions. Thereafter, gene transcription in the cells was quantified using quantitative real-time PCR. Briefly, RNA was extracted from cell lysates using TRI Reagent (Molecular Research Center, Inc., Cincinnati, OH, USA) and quantified by spectrometry at 260 and 280 nm. The RNA was reverse transcribed using SuperScript VILO Master Mix (Thermo Fisher Scientific), and quantitative real-time PCR with cDNA was performed with the StepOnePlus real-time PCR system (Thermo Fisher Scientific) according to manufacturer protocol. TaqMan probes, sense primers, and antisense primers for expression of *GAPDH*, *HLA-DPB1*, *HLA-DQB1*, and *HLA-DRA* were purchased from Thermo Fisher Scientific.


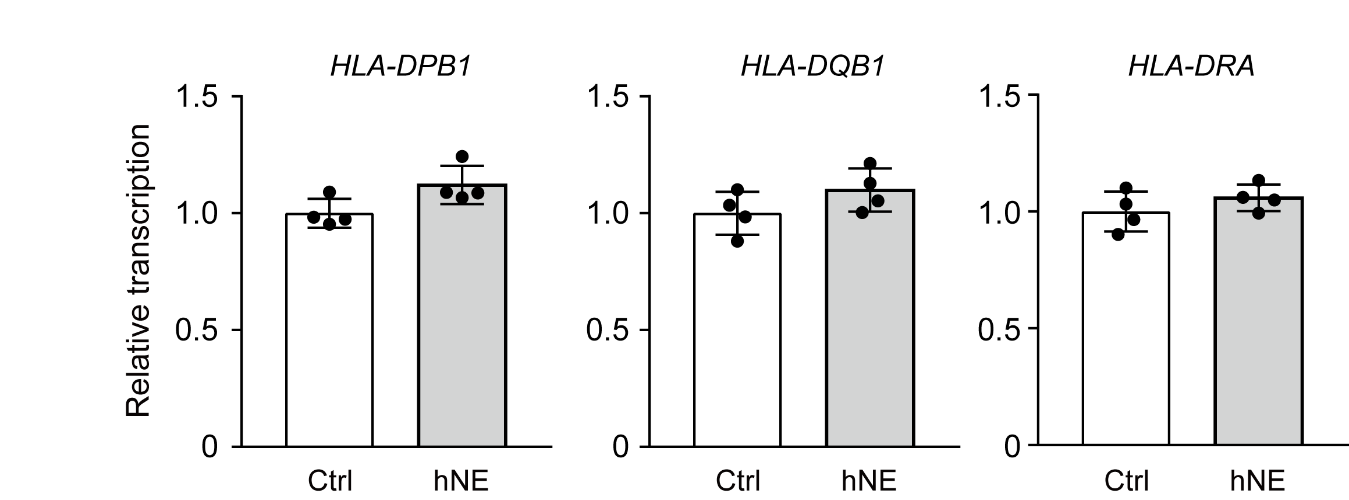


**Supplementary Figure S1. hNE-treatment did not alter transcription of HLA-related genes.**

THP-1-derived macrophages were exposed to 300 mU/mL hNE for 4 h. Real-time PCR was performed to quantify *HLA-DPB1*, *HLA-DQB1*, and *HLA-DRA* mRNA in the cells. The relative quantity of these mRNAs was normalized to the relative quantity of *GAPDH* mRNA. Data representing the mean ± SD of quadruplicate experiments was evaluated by using unpaired *t* tests.


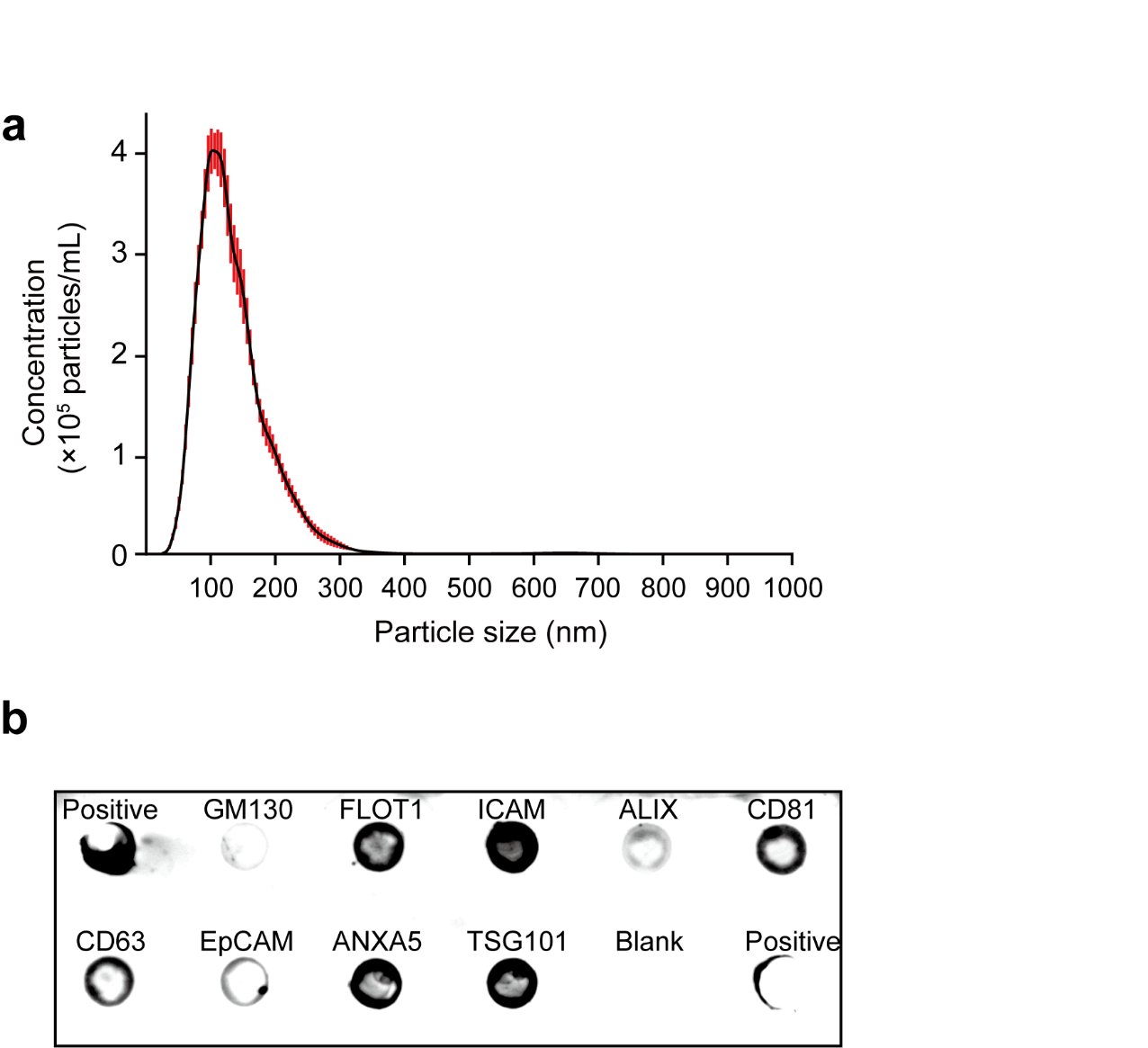


**Supplementary Figure S2. Characterization of extracellular vesicles isolated from THP-1 derived macrophages.**

(A) Particle-size distribution of extracellular vesicles isolated from THP-1 derived macrophages. (B) Quality of extracellular vesicles was examined by using exosome antibody arrays.


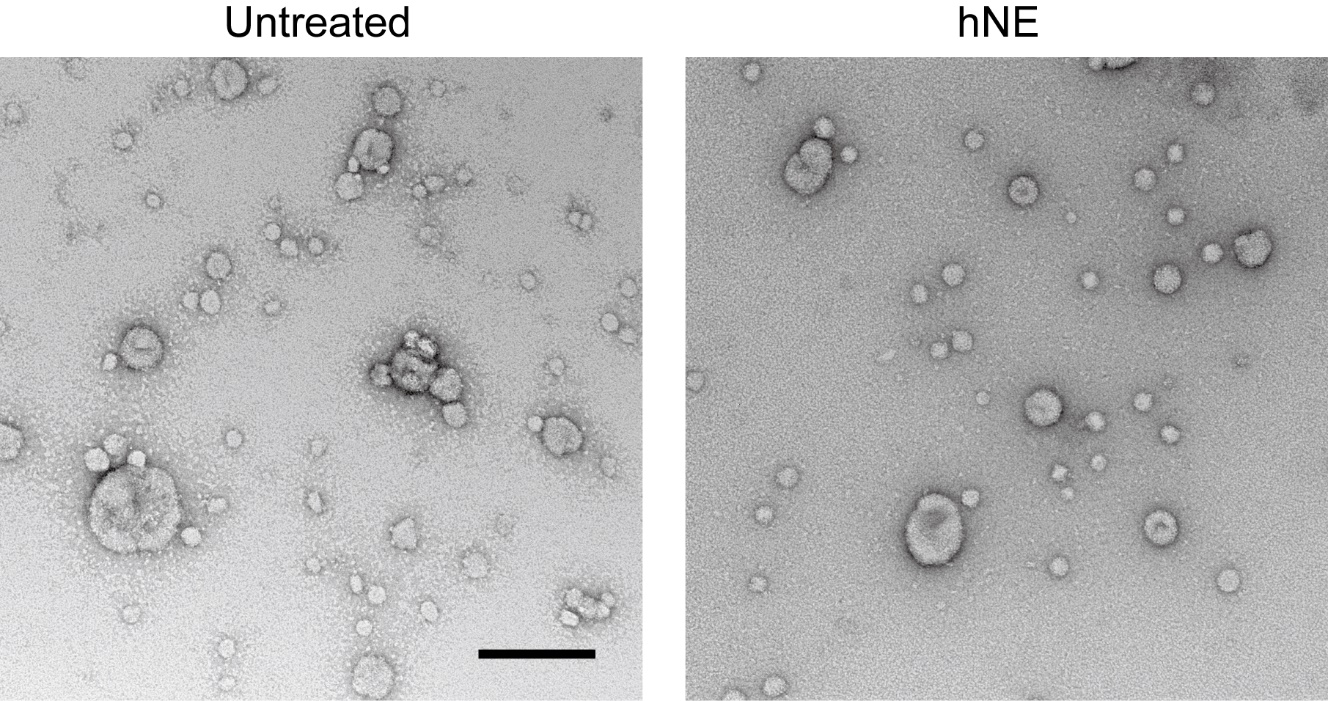


**Supplementary Figure S3. hNE-treatment did not cause morphological changes in extracellular vesicles.**

Extracellular vesicles were isolated with THP-1-derived macrophages followed by treatment with 300 mU/mL human neutrophil elastase (hNE) as described in “Supporting Methods”. Morphological characterization of NE-treated and untreated extracellular vesicles was observed using transmission electron microscopy. Scale bar: 200 nm.


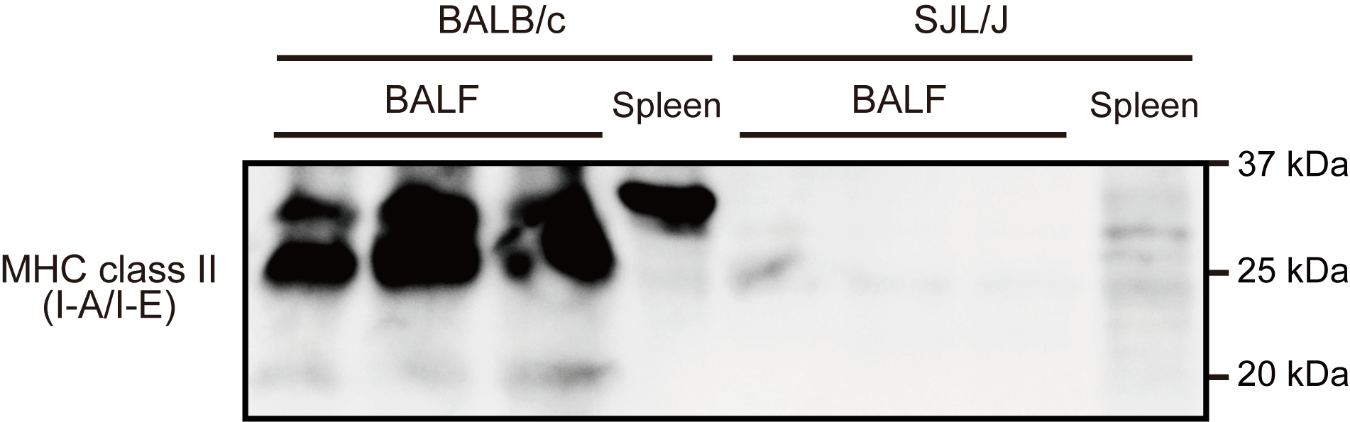


**Supplementary Figure S4. Anti-MHC class II (I-A/I-E) antibody (clone: M5/114) was not specific for bronchoalveolar lavage fluid (BALF) from** **SJL/J mice upon intratracheal infection with pneumococcus.**

BALB/c mice and SJL/J mice were intratracheally infected with *Streptococcus pneumoniae* D39 (2 × 10^8^ colony forming units in 50 µL PBS). Cell-free BALF samples were immunoblotted with anti-MHC class II (I-A/I-E) antibody (clone: M5/114). Mouse spleen lysates were included as controls. A representative western blot image (three samples in each group) was shown.


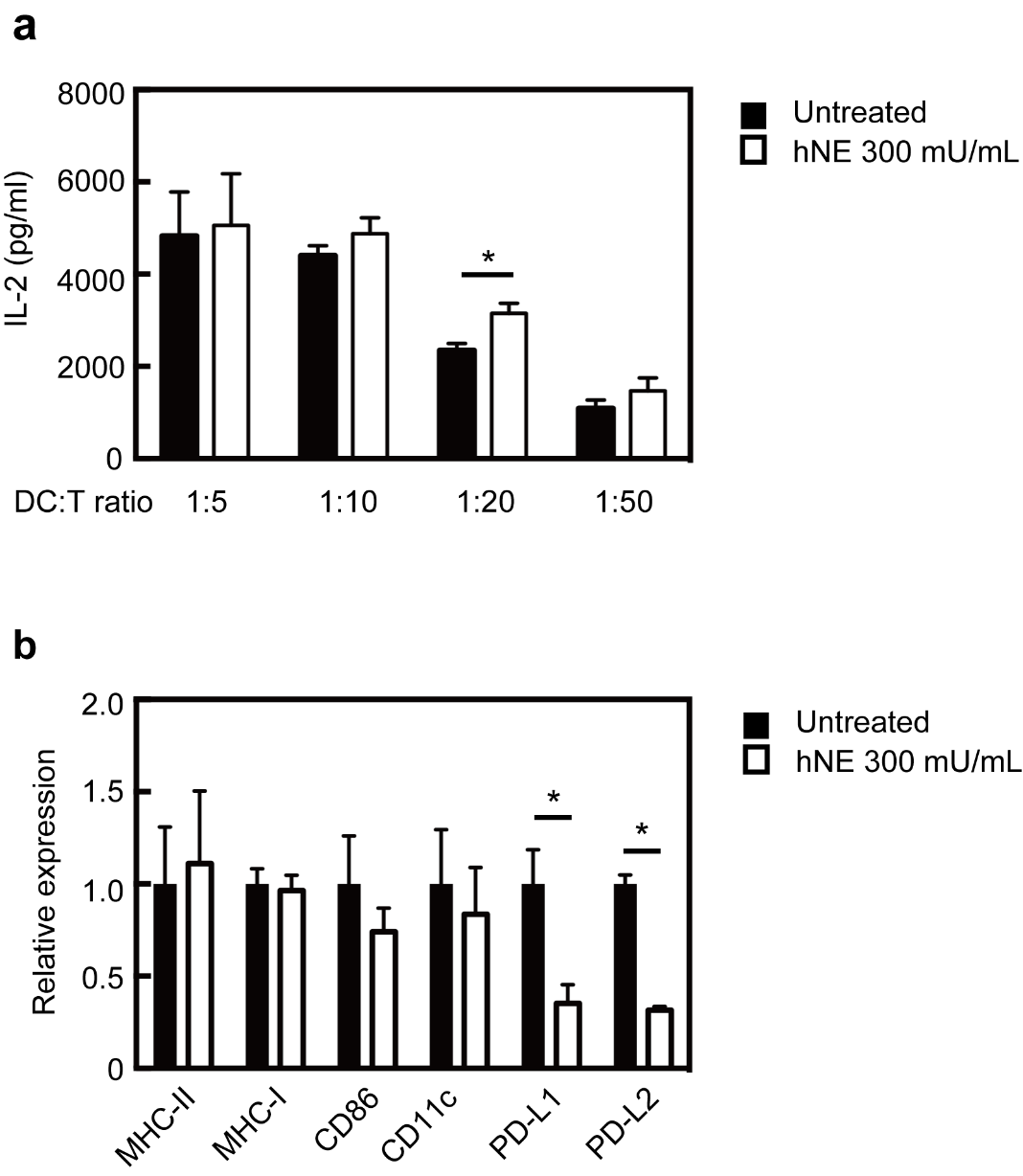


**Supplementary Figure S5. hNE-treatment did not alter mouse MHC class II expression on bone marrow-derived dendritic cells.**

(A) Mouse bone marrow dendritic cells (DC) were incubated with hen egg lysozyme protein followed by treatment with human neutrophil elastase (hNE; 300 mU/mL). Cells were cocultured with 3A9 T cells (T), reactive against hen egg lysozyme protein. Interleukin (IL)-2 concentration in culture supernatants was measured using an ELISA kit. (B) Expressions of mouse major histocompatibility complex (MHC) and co-stimulatory/co-inhibitory molecules on DCs cocultured with 3A9 T cells. Data represent the mean ± SD and were evaluated using unpaired t tests. *Significantly different from untreated group at *P* < 0.05.

**Reference**

1. Inaba, K., Inaba, M., Romani, N., Aya, H., Deguchi, M., Ikehara, S., Muramatsu, S., and Steinman, R. M. (1992) Generation of large numbers of dendritic cells from mouse bone marrow cultures supplemented with granulocyte/macrophage colony-stimulating factor. *J Exp Med* **176**, 1693-1702

**Original panel for each western blot**





 **Figure 2C Figure 2C**

DPβ ̶

GAPDH ̶

DPβ ̶

**Figure 4A Figure 4D**







DPβ ̶

DPβ ̶





**Figure 5 Supplementary Figure S4**
